# Relation of neuropathology with cognitive decline among older persons without dementia

**DOI:** 10.3389/fnagi.2013.00050

**Published:** 2013-09-09

**Authors:** Patricia A. Boyle, Lei Yu, Robert S. Wilson, Julie A. Schneider, David A. Bennett

**Affiliations:** ^1^Rush Alzheimer’s Disease Center, Rush University Medical CenterChicago, IL, USA; ^2^Department of Behavioral Sciences, Rush University Medical CenterChicago, IL, USA; ^3^Department of Neurological Sciences, Rush University Medical CenterChicago, IL, USA; ^4^Department of Pathology, Rush University Medical CenterChicago, IL, USA

**Keywords:** cognitive aging, Alzheimer’s disease, neuropathology, vascular disease, Lewy bodies

## Abstract

**Objective:** Although it is now widely accepted that dementia has a long preclinical phase during which neuropathology accumulates and cognition declines, little is known about the relation of neuropathology with the longitudinal rate of change in cognition among older persons without dementia. We quantified the burden of the neuropathologies of the three most common causes of dementia [i.e., Alzheimer’s disease (AD), cerebrovascular disease (CVD), and Lewy body disease (LBD)] and examined their relation with cognitive decline in a large cohort of persons without dementia proximate to death.

**Methods:** A total of 467 deceased participants without dementia from two longitudinal clinical-pathologic studies, Rush Memory and Aging Project and Religious Orders Study, completed a mean of 7 annual evaluations including 17 cognitive tests. Neuropathologic examinations provided quantitative measures of AD (i.e., amyloid load, tangle density), CVD (i.e., macroscopic infarcts, microinfarcts), and neocortical Lewy bodies. Random coefficient models were used to examine the relation of the neuropathologies with rates of global cognitive decline as well as decline in four specific cognitive systems.

**Results:** At autopsy, 82% of persons without dementia had amyloid, 100% had tangles, 29% had macroscopic infarcts, 25% had microinfarcts, and 6% had neocortical Lewy bodies. Global cognition declined a mean of 0.034 unit per year (SE = 0.003, *p* < 0.001). In separate analyses, amyloid, tangles (*p*-values <0.001) and neocortical Lewy bodies (*p* = 0.015) were associated with an increased rate of global cognitive decline; macroscopic infarcts and microinfarcts were not. Further, when analyzed simultaneously, amyloid, tangles, and neocortical Lewy bodies remained associated with global cognitive decline (*p*-values <0.024). Finally, measures of AD were associated with decline in three of four systems, including episodic memory (i.e., tangles), semantic memory (i.e., amyloid and tangles), and working memory (i.e., amyloid). Lewy bodies also were associated with decline in three of four systems (i.e., semantic memory, working memory, and perceptual speed).

**Interpretation:** The neuropathologies of the common causes of dementia, particularly AD and neocortical LBD, are associated with decline in multiple cognitive abilities among older persons without dementia.

## INTRODUCTION

It is now widely recognized that dementia is characterized by a long preclinical phase during which neuropathology accumulates and cognition declines, and an understanding of the preclinical phase is urgently needed to combat the public health challenge posed by cognitive decline in old age (Sperling et al., 2011). We and others have shown that the neuropathologies of the three most common causes of dementia, Alzheimer’s disease (AD), cerebrovascular disease (CVD), and Lewy body disease (LBD), are frequently observed in the brains of older persons without dementia and related to the level of cognition proximate to death (Crystal et al., 1988; Katzman et al., 1988; Riley et al., 2002; Knopman et al., 2003; Bennett et al., 2006a, 2012c; Sonnen et al., 2007; ). To date, however, few studies have examined the relation of neuropathology with longitudinal rates of cognitive decline among older persons without dementia, and findings are mixed (Green et al., 2000; Galvin et al., 2005; Driscoll et al., 2006; Balasubramanian et al., 2012). Moreover, the studies that examined the relation of neuropathology with cognitive decline focused on a single neuropathology (i.e., AD), despite evidence that mixed neuropathologies are common and are most strongly associated with cognitive impairment (Schneider et al., 2007). An understanding of the relation of the neuropathologies of the common causes of dementia with longitudinal rates of cognitive decline among persons without dementia has implications for efforts to reduce the public health burden posed by cognitive decline in old age.

In this study, we examined the relation of the neuropathologies of AD, CVD, and LBD, with longitudinal rates of cognitive decline among 467 older persons who were free of dementia at the time of death. Participants were from two large cohort studies of aging, the Rush Memory and Aging Project and the Religious Orders Study, and had detailed annual cognitive function data available for up to 18 years (Bennett et al., 2012a,b). All underwent autopsies that yielded quantitative measures of multiple neuropathologies, including measures of AD (i.e., amyloid load, tangle density), CVD (i.e., macroscopic infarcts, microinfarcts), and neocortical Lewy bodies (Bennett et al., 2006a, 2012c; Arvanitakis et al., 2011; Schneider et al., 2012). Random coefficient models were used to characterize linear rates of cognitive decline and examine the relation of the neuropathologies with decline in global cognition and four specific cognitive systems (Laird and Ware, 1982).

## MATERIALS AND METHODS

### PARTICIPANTS

Participants were from two clinical–pathologic cohort studies of aging, the Religious Orders Study and the Memory and Aging Project (Bennett et al., 2012a,b). The Religious Orders Study began in 1994 and involves older Catholic nuns, priests, and monks from more than 40 groups across the United States. The Rush Memory and Aging Project began in 1997 and involves older lay persons from retirement communities and subsidized housing facilities in the Chicago metropolitan area. All participants agreed to annual clinical evaluations and brain autopsy at death. Written informed consent was obtained in each study after procedures were fully explained, and both studies were approved by the Institutional Review Board of Rush University Medical Center. The follow up participation rates exceed 95% of survivors and autopsy rates exceed 80%. At the time of these analyses, data were available from 467 non-demented deceased persons with at least two cognitive evaluations.

### CLINICAL AND COGNITIVE EVALUATION PROCEDURES

Participants in both studies underwent structured, annual clinical evaluations that included detailed cognitive and neurologic examinations, as previously reported (Bennett et al., 2012a,b). The two studies have 19 cognitive tests in common. Scores from 17 of those tests were converted to z scores using the mean and standard deviation of the cohort at baseline, and z-scores were averaged to yield a summary measure of global cognition (Bennett et al., 2012a,b,c). The use of a composite score has the advantage of minimizing floor and ceiling effects and other sources of random variability and is preferable for studies examining longitudinal rates of change in cognition.

### BRAIN AUTOPSY PROCEDURES AND QUANTIFICATION OF PATHOLOGY MEASURES

Brains were removed, weighed, and immersion fixed in 4% paraformaldehyde for at least 72 h. One centimeter coronal slabs of brain from both hemispheres were grossly inspected and pathology was recorded, as previously described (Bennett et al., 2006a, 2012a,b,c; Arvanitakis et al., 2011; Schneider et al., 2012). After gross examination, nine brain regions of interest (i.e., midfrontal, midtemporal, inferior parietal, anterior cingulate, entorhinal and hippocampal cortices, basal ganglia, thalamus, and midbrain) were dissected from the 1-cm slabs of fixed tissue and processed and embedded in paraffin. Sections (6 μm) from the paraffin blocks were stained with hematoxylin and eosin for general pathology including microscopic infarcts. In addition, multiple tissue blocks from eight brain regions (i.e., entorhinal cortex, CA1/subiculum of the hippocampus, superior frontal cortex, dorsolateral prefrontal cortex, inferior temporal cortex, angular gyrus, anterior cingulate cortex, and occipital cortex) were cut into 20 μm sections to immunohistochemically identify and quantify amyloid load and PHFtau positive tangle density.

Amyloid-β was labeled with an N-terminus–directed monoclonal antibody in eight brain regions (described above; 10D5; Elan, Dublin, Ireland; 1:1,000), as previously described (Schneider et al., 2007, 2012; Arvanitakis et al., 2011). Immunohistochemistry was performed using diaminobenzidine as the reporter, with 2.5% nickel sulfate to enhance immunoreaction product contrast. Briefly, the brain region of interest was outlined at low power using StereoInvestigator software version 9 (MicroBrightfield, Colchester, VT, USA) and an Olympus (Tokyo, Japan) BX-51 microscope with an attached motorized stage. A grid was randomly placed over the outlined area. Following camera and illumination calibration, the magnification was raised to the 20× objective, and images at each sampling site were obtained with a motorized stage. Quantification of amyloid-β load was accomplished by image processing in an automated, multistage computational image analysis protocol. Mean fraction (percentage area positive for amyloid) per brain region and per subject was computed.

PHFtau was labeled with an antibody specific for phosphorylated tau (AT8; Innogenetics, San Ramon, CA, USA; 1:1,000) in all eight brain regions. Quantification of tangle density was accomplished with the stereological mapping station. Briefly, after the brain region of interest was delimited at low power, a grid was randomly placed over the entire brain region by the software program. Magnification was raised to the 40× objective, and the program was engaged to direct the motorized stage to stop at each intersection point of the grid for sampling. All objects within the 150 μm × 150 μm counting frame that did not touch the exclusion lines of the box (bottom and left sides) were counted. Quantification of tangle density was done via the stereological mapping station and yielded a measure of the density of tangles (per mm^2^) that was averaged within and across brain regions.

For each brain, the age, volume (in cubic millimeters), and anatomic location of all macroscopic cerebral infarctions were identified in both hemispheres. All macroscopic infarcts were dissected, paraffin embedded and cut and stained, allowing for confirmation of the lesion. Age and locations of old microinfarcts, defined as infarcts seen on microscopy but not identified on gross examination, were based on analysis of sections from nine regions of a single hemisphere.Macroscopic and microscopic infarctions were analyzed as dichotomous variables (i.e., present/absent; Riley et al., 2002; Schneider et al., 2007, 2012).

Neocortical Lewy bodies were identified with an antibody to α-synuclein, and only intracytoplasmic Lewy bodies were used as an indicator of positive staining. To simplify criteria for the different types of Lewy body pathology, the McKeith criteria were modified such that nigral predominant Lewy body pathology included cases with Lewy bodies in the substantia nigra without evidence of Lewy bodies in the limbic or neocortical regions. Limbic-type LBD included cases with either anterior cingulate or entorhinal positivity (typically also with nigral pathology) without neocortical Lewy body pathology. Finally neocortical-type Lewy body pathology required Lewy bodies in either midfrontal, temporal, or inferior parietal cortex with either nigral or limbic positivity, but often with both. Each case was classified as 0 = no Lewy bodies, 1 = nigral predominant, 2 = limbic-type, or 3 = neocortical-type Lewy body pathology. Neocortical Lewy body pathology was used in these analyses as a dichotomous variable (i.e., present/absent) given the strong association of neocortical Lewy body pathology with cognition.

### STATISTICAL ANALYSES

We first describe the characteristics of the participants. Next, we used random coefficient models to characterize linear rates of cognitive decline and examine the relation of neuropathology with cognitive decline (Laird and Ware, 1982). We began with an initial model with terms for age, sex, education, time, and the interactions of time with the demographic variables; time is defined as the time (in years) prior to death. The estimate for time (i.e., the slope) corresponds to the mean rate of decline in cognition. We then added in terms for the neuropathologies and their interactions with time in a series of models. Each significant time-by-neuropathology term characterizes the difference from the mean slopes of cognitive decline associated with the neuropathology studied. The core (fully adjusted) model included terms for age, sex, education, time, all of the neuropathologies, and the interactions of time with all demographic and neuropathologic variables. Finally, because it is possible that regionally specific neuropathology may have differential effects on cognitive decline among persons without dementia, we next examined the relation of the neuropathologies with decline in cognition in a series of models examining mesial temporal and neocortical amyloid and tangles, respectively; the measures of amyloid and tangles were examined in separate models given their high correlations (Spearman r for mesial temporal and neocortical amyloid = 0.91, *p* < 0.001 and for mesial temporal and neocortical tangles = 0.61, *p* < 0.001) and all analyses controlled for the presence of CVD and LBD. Analyses were performed in SAS®9.2(SAS Institute Inc., Cary, NC, USA).

## RESULTS

### DESCRIPTIVES

Participants (*n* = 467) in these analyses completed a mean of 7.0 (SD = 3.7, range = 2–18) years of annual cognitive function testing and were an average of 87 (SD = 6.5, range = 66–106) years of age at the time of death; additional descriptive data are provided in **Table 1**. The distribution of the global cognitive measure at baseline was approximately normal (mean = 0.07, SD = 0.45). At autopsy, 382 (82%) of persons were positive for amyloid, 467 (100%) were positive for tangles, 133 (29%) had one or more macroscopic infarcts, 117 (25%) had one or more microinfarcts, and 29 (6%) had neocortical Lewy bodies.

**Table 1:** Descriptive characteristics of the cohort.

Variable	Mean, SD, range (or # and percent)
Age at death	87, 6.5, 66–106
Education	16.2, 3.6, 5–30
% Female	295, 63%
% White, non-Hispanic	446, 96%
Baseline MMSE	28.2, 1.8, 18–30
Proximate to death MMSE	27, 3.1, 0–30
Baseline global cognition	0.07, 0.45, -1.85 to 1.38
Proximate to death global cognition	-0.14, 0.55, –2.45 to 1.28
Amyloid	3.16, 3.93, 0–22.9
Tangles	3.72, 4.22, 0–35.2
Gross infarcts (1+ present)	133, 29%
Microinfarcts (1+ present)	117, 25%
Neocortical Lewy bodies (present)	29, 6%

### HETEROGENEITY OF AGE-RELATED COGNITIVE DECLINE AMONG OLDER PERSONS WITHOUT DEMENTIA

**FIGURE 1:**
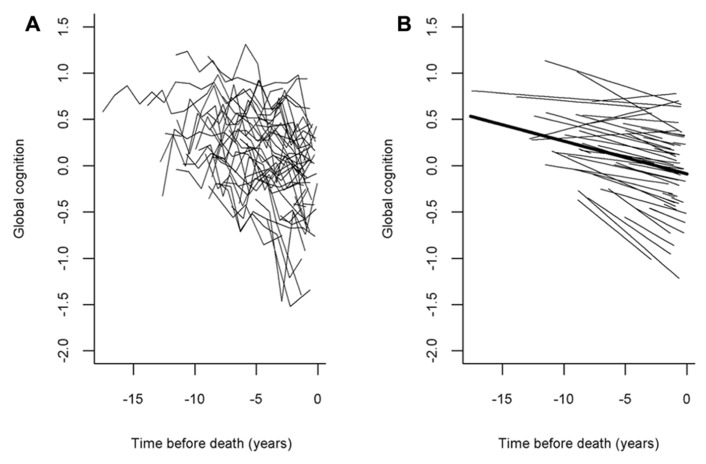
**The panel(A) shows a spaghetti plot of individual trajectories from a random sample of persons (*N* = 50, raw data points plotted for each individual) and the panel(B) shows the mean slope of cognitive decline superimposed on their estimated individual slopes (model-derived)**.

We used random coefficient models to characterize the annual rate of decline (slope) in cognition; the primary outcome was the longitudinal rate of change in global cognition, and this and all subsequent analyses adjusted for age, sex, and education. In the initial model, the global cognitive measure declined a mean of about 0.034 unit per year (SE = 0.003, *p* = 0.0014). **Figure 1** shows a spaghetti plot of raw data points for a random sample (*N* = 50) of these participants **Figure 1A** as well as the model-derived mean linear slope of cognitive decline (**Figure 1B**, dark line) superimposed on the estimated individual slopes from the 50 individuals whose raw data points are shown. As the figure illustrates, most persons exhibited decline, but there was considerable heterogeneity in person-specific slopes.

### RELATION OF AGE-RELATED NEUROPATHOLOGIES WITH THE RATE OF GLOBAL COGNITIVE DECLINE

We first examined the relation of each neuropathology with the rate of global cognitive decline in separate models. In the initial analysis, amyloid was associated with a faster rate of cognitive decline, such that a one percent increase in amyloid was associated with a 0.002 unit (SE = 0.0006, *p* < 0.001) faster rate of decline in global cognition. Tangles (estimate = -0.003, SE = 0.0006, *p* < 0.001) and neocortical Lewy bodies (estimate = -0.024, SE = 0.009, *p* = 0.015) also were associated with a faster rate of decline. Macroscopic and microinfarcts were not significantly associated with the rate of decline.

**FIGURE 2:**
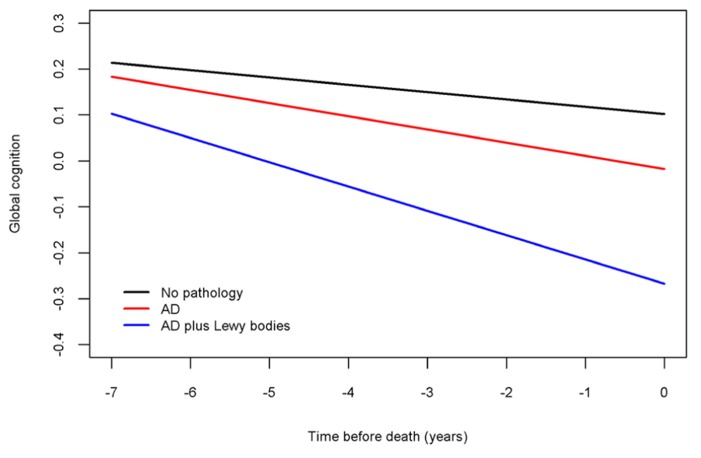
**Contributions of combinations of the neuropathologies to cognitive decline (model-derived slopes; top line shows the minimal decline associated with no pathology, the red line shows the increased rate of decline associated with AD pathology, and the blue line shows the increased rate of decline associated with LBD pathology)**.

**FIGURE 3:**
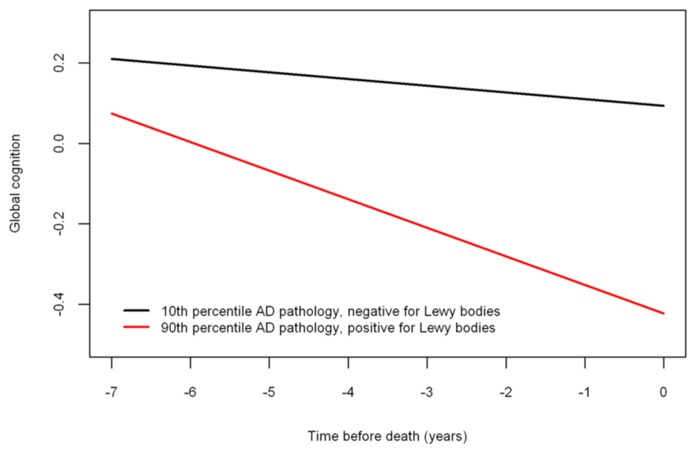
**Relation of neuropathology to cognitive decline (model-derived slopes) among a typical participant with a low burden of pathology (10th percentile amyloid = 0 and tangles = 0.311 and negative for neocortical LBD) versus a participant with a high burden of pathology (90th percentile amyloid = 9.262 and tangles = 8.048 and positive for neocortical LBD)**.

Next, because previous work suggests that the neuropathologies of the three most common causes of dementia frequently co-occur and mixed pathologies are most strongly associated with cognitive decline, we examined the relation of all of the neuropathologies simultaneously with the slope of global cognitive decline. When considered together, amyloid (estimate = -0.0014, SE = 0.0006, *p* < 0.023), tangles (estimate = -0.0023, SE = 0.0006, *p* < 0.001), and neocortical Lewy bodies (estimate = -0.0243, SE = 0.0096, *p* = 0.011) remained associated with faster rates of decline. **Figure 2** is based on the fully adjusted model and illustrates the additive effect of these neuropathologies on the rate of cognitive decline. This figure shows that AD pathology (i.e., the mean of amyloid and tangles) is associated with an increased rate of decline compared to the very modest decline associated with no pathology, but the rate of decline is greatest for persons with both AD pathology and neocortical Lewy bodies. **Figure 3** further illustrates the relation of the severity of pathology with cognitive decline.

### RELATION OF AGE-RELATED NEUROPATHOLOGIES WITH DECLINE IN FOUR COGNITIVE SYSTEMS

Next, because it is possible that the neuropathologies of AD, CVD, and LBD have differential effects on specific cognitive systems among persons without dementia, we next examined the relation of the neuropathologies with rates of decline in four specific cognitive systems (i.e., episodic memory, semantic memory, working memory, and perceptual speed). In models considering all neuropathologies together, measures of AD were related to decline in three of the four systems, including episodic memory (i.e., tangles), semantic memory (i.e., amyloid, tangles), and working memory (i.e., amyloid), and neocortical Lewy bodies were related to semantic memory, working memory, and perceptual speed (**Table 2**). Macroscopic infarcts were related to decline in semantic memory only, and microinfarcts were not related to decline in any of the cognitive systems.

**Table 2:** Relation of the neuropathologies with rate of decline in four cognitive systems^*^.

Term	Estimate (SE) *p*
**Episodic memory**
Tangles	–0.0045 (0.0009) *p* < 0.001
Amyloid	Ns
Gross infarcts	Ns
Microinfarcts	Ns
Neocortical Lewy bodies	Ns
**Semantic memory**
Tangles	–0.0031 (0.0006) *p* < 0.001
Amyloid	–0.0012 (0.0006) *p* = 0.044
Gross infarcts	–0.0139 (0.0057) *p* = 0.016
Microinfarcts	Ns
Neocortical Lewy bodies	–0.0303 (0.0094) *p* = 0.001
**Working memory**
Tangles	Ns
Amyloid	–0.0021 (0.0007) *p* = 0.004
Gross infarcts	Ns
Microinfarcts	Ns
Neocortical Lewy bodies	–0.0221 (0.0109) *p* = 0.042
**Perceptual speed**
Tangles	Ns
Amyloid	Ns
Gross infarcts	Ns
Microinfarcts	Ns
Neocortical Lewy bodies	–0.0784 (0.0160) *p* < 0.001

* Derived from random coefficient models including terms for age, sex, education, time, all of the neuropathologies, and the interactions of demographics and the neuropathologies with time.

### RELATION OF REGIONALLY SPECIFIC NEUROPATHOLOGY WITH COGNITIVE DECLINE

Finally, because it is possible that regionally specific neuropathology may have differential effects on cognitive decline among persons without dementia, we next examined the relation of the neuropathologies with decline in a series of models examining mesial temporal and neocortical amyloid and tangles, respectively; all analyses controlled for the presence of CVD and LBD. Both mesial temporal (estimate = -0.0027, SE = 0.0009, *p* = 0.002) and neocortical amyloid (estimate = -0.0019, SE = 0.0005, *p* < 0.001) were related to global cognitive decline, as were mesial temporal (estimate = -0.0008, SE = 0.0002, *p* < 0.001) and neocortical tangles (estimate = -0.0037, SE = 0.0010, *p* < 0.001). Mesial temporal and neocortical amyloid and tangles also were related to decline in episodic memory and semantic memory, but only amyloid was related to decline in working memory (both mesial temporal and neocortical amyloid) and perceptual speed (mesial temporal only; **Table 3**).

**Table 3:** Relation of the regionally specific neuropathology with rate of decline in four cognitive systems^*^.

Term	Estimate (SE) *p*
**Episodic memory**
Mesial temporal amyloid	-0.0028 (0.0012) *p* = 0.025
Neocortical amyloid	-0.0020 (0.0008) *p* = 0.0111
Mesial temporal tangles	-0.0015 (0.0003) *p* < 0.001
Neocortical tangles	-0.0059 (0.0014) *p* < 001
**Semantic memory**
Mesial temporal amyloid	-0.0026 (0.0009) *p* = 0.004
Neocortical amyloid	-0.0019 (0.0006) *p* = 0.001
Mesial temporal tangles	-0.0009 (0.0002) *p* < 0.001
Neocortical tangles	-0.0053 (0.0010) *p* < 0.001
**Working memory**
Mesial temporal amyloid	-0.0029 (0.0010) *p* = 0.003
Neocortical amyloid	-0.0017 (0.0006) *p* = 0.007
Mesial temporal tangles	Ns
Neocortical tangles	Ns
**Perceptual speed**
Mesial temporal amyloid	-0.0031 (0.0014) *p* = 0.003
Neocortical amyloid	Ns
Mesial temporal tangles	Ns
Neocortical tangles	Ns

* Derived from separate random coefficient models including terms for age, sex, education, time, CVD, LBD, the relevant regionally specific measure of pathology, and the interactions of demographics and the pathologies with time.

## DISCUSSION

We examined the burden and cognitive consequences of the neuropathologies of the three most common causes of dementia in old age, AD, CVD, and LBD, in a cohort of more than 450 deceased persons who were free of dementia at the time of death and had detailed annual cognitive assessments for up to 18 years prior to death. AD pathology was present in the majority of persons, about a third had evidence of CVD, and a relatively small number had neocortical Lewy bodies. Further, AD pathology and neocortical Lewy bodies were associated with an increased rate of decline in global cognition, as well as multiple cognitive systems. These findings suggest that the neuropathologies of the common causes of dementia, particularly AD and neocortical LBD, are important drivers of cognitive decline even among persons without dementia.

Prior studies have shown that the neuropathologies of the common causes of dementia are frequently found in the brains of older persons without dementia. For example, upward of 30% of older persons who die without a diagnosis of clinical dementia meet pathologic criteria for AD, and the presence of pathologic AD is related to cognitive decrements, particularly in episodic memory (Crystal et al., 1988; Katzman et al., 1988; Riley et al., 2002; Knopman et al., 2003; Bennett et al., 2006a). Further, we and others have shown that the neuropathologies of AD (measured continuously as done here), CVD, and LBD are common among persons without dementia and even among persons without any cognitive impairment (i.e., no dementia or mild cognitive impairment) and are related to the level of cognitive function proximate to death (Riley et al., 2002; Knopman et al., 2003; Bennett et al., 2006a, 2012c). However, prior work has not elucidated the relation of these neuropathologies with longitudinal rates of change in cognition among persons who die without dementia. The studies that examined the relation of neuropathology with cognitive decline in persons without dementia typically used brief cognitive assessments and/or had relatively small samples with short lengths of follow-up, and all focused on a single pathology (e.g., AD, typically using Braak or other staging methods rather than continuous measures as used here). The latter is a particularly important limitation, given that mixed pathologies are common and cognitive impairment in old age is most often due to mixed neuropathologies (Green et al., 2000; Galvin et al., 2005; Driscoll et al., 2006; Schneider et al., 2007; Balasubramanian et al., 2012). Here, we extended prior work in three important ways: first, we examined the relation of neuropathology with the rate of cognitive decline in global cognition measured using 17 cognitive tests, as well as four specific cognitive systems; second, we studied a relatively large cohort of older persons with repeated cognitive assessments for up to 18 years; third, we examined multiple neuropathologic indices of the three most common causes of dementia in old age, AD, CVD, and neocortical LBD. We found that the neuropathologies of AD and neocortical LBD were associated with a faster rate of decline in global cognition and multiple cognitive systems among persons who died without dementia.

An understanding of the extent to which common neuropathologies contribute to preclinical changes in cognition is needed to facilitate efforts to prevent or delay the earliest manifestations of cognitive decline. Even very early and mild forms of cognitive decline have important functional consequences; for example, we have previously shown that cognitive decline among persons without dementia or even mild cognitive impairment is associated with impaired decision making and increased susceptibility to scams (Boyle et al., 2012b). The present results support the notion that cognitive decline begins well before the onset of dementia and is related to the accumulation of the common neuropathologies known to cause dementia. Further, multiple cognitive abilities are affected by these neuropathologies, but there was little evidence of regionally specific associations; that is, both mesial temporal and neocortical amyloid and tangles were related to global cognitive decline. In addition, whereas both mesial temporal and neocortical amyloid and tangles were related to decline in the domains of episodic memory and semantic memory, mesial temporal and neocortical amyloid were related to decline in working memory, and mesial temporal amyloid was related to perceptual speed. The lack of regional associations is consistent with the idea that cognitive abilities tend to become increasingly correlated in late life as pathology accumulates (Boyle et al., 2013) and is not surprising given that most of the data showing region-specific associations come from lesion studies in which there is damage to well-demarcated brain areas from organisms with otherwise healthy brains. In addition, the regional measures of the pathology are relatively highly correlated. Thus, in the context of age-related cognitive decline, which typically involves multiple related neuropathologies, the association of pathology with cognition may be of a more general nature.

The present findings suggest that at least some of the cognitive decline observed among non-dementeds, that which is sometimes considered “benign”, is in fact driven by pathologic processes and highlight the important role the common neuropathologies play in the early, preclinical phase of cognitive decline; thus, these data underscore the need for therapies that can be utilized at the earliest manifestation of symptoms or earlier, before irreversible damage occurs. Identification of the factors slow the rate of cognitive decline in the face of accumulating neuropathology is urgently needed. To date, scientific efforts have focused much less on the factors that protect against cognitive decline and dementia than on factors that predict dementia and other negative health outcomes, and our understanding of the mechanisms that allow some persons to remain dementia-free despite accumulating neuropathology is still in its infancy. Several positive lifestyle (e.g., participation in cognitive, physical, and social activities) and psychosocial factors (e.g., extraversion, purpose in life, lifespace) have been shown to be protective against the risk of dementia and rate of cognitive decline (Wilson et al., 2002, 2007; Bennett et al., 2006b; Boyle et al., 2010, 2012a; James et al., 2011; Buchman et al., 2012; Wilson et al., 2012). Some of these (e.g., purpose in life, social networks) modify the association of AD pathology with cognitive function, such that persons high in the protective factor function better cognitively despite the burden of neuropathology (Bennett et al., 2006b; Boyle et al., 2012a). However, the neurobiologic mechanisms underlying the protective effects of these lifestyle and psychosocial factors have yet to be identified. The concept of cognitive or neural reserve posits that individual differences in the ability to tolerate or respond to accumulating pathology affect the degree to which pathologic processes are expressed as decline or impairment (Stern, 2002). Given that the neuropathologies of AD, CVD, and neocortical LBD were relatively frequent in the non-demented persons included in this study, reserve may somehow have protected them from developing overt dementia. In keeping with this idea, in a prior study, we found that neuronal density in the locus coeruleus was associated with a reduced rate of cognitive decline after controlling for AD, CVD, and LBD (Wilson et al., 2013). We also found that presynaptic proteins were associated with better cognition proximate to death and a slower rate of cognitive decline after controlling for AD, CVD, and LBD (Honer et al., 2012; Boyle et al., 2013). Increased research focus is needed to elucidate the mechanisms by which indices of reserve operate to protect against cognitive decline and dementia in the face of accumulating neuropathology.

The study has strengths and limitations. Strengths are that all subjects were recruited from the community and known to be free of dementia proximate to death, all underwent detailed annual cognitive evaluations for up to 18 years, and autopsy rates were very high. In addition, all post-mortem evaluations were performed by experienced and trained examiners shielded to all clinical data and multiple biologically specific measures of pathology were quantified. The study also has limitations. For example, the findings are from a selected cohort and their generalizability remains to be demonstrated. Future work is needed to better understand the impact of the common causes of dementia as well as protective factors that influence cognition in population-based studies of older persons who remain free of dementia throughout life.

## Conflict of Interest Statement

The authors declare that the research was conducted in the absence of any commercial or financial relationships that could be construed as a potential conflict of interest.

## AUTHOR CONTRIBUTIONS

Patricia A. Boyle had full access to all of the data in the study and takes responsibility for the integrity of the data and the accuracy of the data analysis.

**Study concept and design:** David A. Bennett, Patricia A. Boyle, Julie A. Schneider, Robert S. Wilson. **Acquisition of data:** David A. Bennett, Julie A. Schneider. **Analysis and interpretation of data:** Patricia A. Boyle, David A. Bennett, Robert S. Wilson, Lei Yu. **Drafting of the manuscript:** Patricia A. Boyle. **Critical revision of the manuscript for important intellectual content:** Robert S. Wilson, Lei Yu, David A. Bennett, Julie A. Schneider. **Statistical analysis:** Lei Yu. **Obtained funding:** David A. Bennett, Patricia A. Boyle. **Study supervision:** David A. Bennett, Julie A. Schneider.

## ROLE OF THE SPONSORS

National Institute on Aging and the Illinois Department of Public Health had no role in the design and conduct of the study or in the collection, analysis, and interpretation of the data and preparation, review, and approval of the manuscript.
